# Purification, Characterization and Bactericidal Action of Lysozyme, Isolated from *Bacillus subtillis* BSN314: A Disintegrating Effect of Lysozyme on Gram-Positive and Gram-Negative Bacteria

**DOI:** 10.3390/molecules28031058

**Published:** 2023-01-20

**Authors:** Muhammad Naveed, Yadong Wang, Xian Yin, Malik Wajid Hussain Chan, Sadar Aslam, Fenghuan Wang, Baocai Xu, Asad Ullah

**Affiliations:** 1 Beijing Advanced Innovation Center for Food Nutrition and Human Health, Beijing Technology & Business University (BTBU), Beijing 100048, China; 2 School of Light Industry, Beijing Technology & Business University (BTBU), Beijing 100048, China; 3 Department of Chemistry, Faculty of Science, Federal Urdu University of Arts, Science and Technology, Campus Gulshan-e-Iqbal, Karachi 75300, Pakistan; 4 Department of Biological Science, University of Baltistan, Skardu 16400, Pakistan; 5 Food and Marine Resources Research Center, Pakistan Council of Scientific and Industrial Research Laboratories Complex, Karachi 75280, Pakistan

**Keywords:** *Bacillus subtilis* BSN314, lysozyme purification, enzymatic activity, antibacterial activity, atomic force microscopy, SDS-PAGE, LC-MS

## Abstract

In the present study, lysozyme was purified by the following multi-step methodology: salt (ammonium sulfate) precipitation, dialysis, and ultrafiltration. The lysozyme potential was measured by enzymatic activity after each purification step. However, after ultrafiltration, the resulting material was considered extra purified. It was concentrated in an ultrafiltration centrifuge tube, and the resulting protein/lysozyme was used to determine its bactericidal potential against five bacterial strains, including three gram-positive (*Bacillus subtilis* 168, *Micrococcus luteus*, and *Bacillus cereus*) and two gram-negative (*Salmonella typhimurium* and *Pseudomonas aeruginosa*) strains. The results of ZOI and MIC/MBC showed that lysozyme had a higher antimicrobial activity against gram-positive than gram-negative bacterial strains. The results of the antibacterial activity of lysozyme were compared with those of ciprofloxacin (antibiotic). For this purpose, two indices were applied in the present study: antimicrobial index (AMI) and percent activity index (PAI). It was found that the purified lysozyme had a higher antibacterial activity against *Bacillus cereus* (AMI/PAI; 1.01/101) and *Bacillus subtilis* 168 (AMI/PAI; 1.03/103), compared to the antibiotic (ciprofloxacin) used in this study. Atomic force microscopy (AFM) was used to determine the bactericidal action of the lysozyme on the bacterial cell. The purified protein was further processed by gel column chromatography and the eluate was collected, its enzymatic activity was 21.93 U/mL, while the eluate was processed by native-PAGE. By this analysis, the un-denatured protein with enzymatic activity of 40.9 U/mL was obtained. This step shows that the protein (lysozyme) has an even higher enzymatic potential. To determine the specific peptides (in lysozyme) that may cause the bactericidal potential and cell lytic/enzymatic activity, the isolated protein (lysozyme) was further processed by the SDS-PAGE technique. SDS-PAGE analysis revealed different bands with sizes of 34 kDa, 24 kDa, and 10 kDa, respectively. To determine the chemical composition of the peptides, the bands (from SDS-PAGE) were cut, enzymatically digested, desalted, and analyzed by LC-MS (liquid chromatography-mass spectrometry). LC-MS analysis showed that the purified lysozyme had the following composition: the number of proteins in the sample was 56, the number of peptides was 124, and the number of PSMs (peptide spectrum matches) was 309. Among them, two peptides related to lysozyme and bactericidal activities were identified as: A0A1Q9G213 (N-acetylmuramoyl-L-alanine amidase) and A0A1Q9FRD3 (D-alanyl-D-alanine carboxypeptidase). The corresponding protein sequence and nucleic acid sequence were determined by comparison with the database.

## 1. Introduction

Lysozyme is an enzyme also known as muramidase, 1,4-β-N-acetylmuramidase, or N-acetylmuramide glycanhydrolase. It is ubiquitous in animals, plants, and microorganisms [1,2], and is commonly found in various human body secretions, such as tears, saliva, blood serum, milk, and urine. It is also a necessary component of avian egg-white [3]. It is an enzyme that has a bactericidal potential against various pathogenic bacteria. During its bactericidal activity, it biodegrades the bacterial cell wall by catalyzing the hydrolysis of the β-(1,4)-glycosidic bonds between N-acetylmuramic acid and N-acetylglucosamine in the peptidoglycan layer, which is the main component of the cell wall of both gram-positive and gram-negative bacteria [4]. Lysozyme has six major types, e.g., phage-lysozyme, bacterial-lysozyme, c(chicken)-type, g(goose)-type, I(invertebrate)-type, and plant lysozyme [5]. Bacterial lysozyme appears to be very similar to egg-white lysozyme [4,6,7]. It consists of 129 amino acids (monomeric protein), stabilized by four disulfide bonds between the eight cysteine molecules of its chain [8,9]. This biochemical structure of lysozyme enhances its bactericidal potential by breaking down the bacterial cell wall. Because of this ability, lysozyme is a naturally occurring potent antibiotic and is considered as an innate potent antibiotic for defense against various microbes [9,10]. The effect of lysozyme against different types of bacteria is not the same, e.g., it has a higher bactericidal potential against gram-positive than against gram-negative bacteria. This is because gram-positive bacteria have a thick peptidoglycan layer and no outer lipid membrane, whereas gram-negative bacteria have a thin peptidoglycan layer and an outer lipid layer. The lipid layer provides maximum protection to gram-negative bacteria; therefore, gram-positive bacteria are easily killed by lysozyme [11,12,13]. 

Food quality plays a very important role in the world, because substandard food has caused public health problems all over the world. There are many diseases that are spread by unsafe food. They range from diarrhea to various forms of cancer and foodborne diseases. These diseases cause enormous socioeconomic problems for societies [10,14,15,16,17]. In this context, preservation methods play an important role in maintaining food quality in the industry. However, most of these methods are not safe because they affect the food quality through microbial, physical, and chemical deterioration. Foods that are preserved in unhealthy ways have a direct impact on consumer health. In the last decade, researchers have attempted to introduce safe preservation methods into the food industry, and because they have no side effects, bio-preservation methods have been widely used and recommended to the industry. These methods maintain the quality of food by preserving its original texture [18,19,20,21,22]. The use of microorganisms, such as bacteria, fungi, yeasts, etc., as bio-preservatives are considered safe for maintaining food quality because their antimicrobial activity inhibits the growth of pathogens in food. This method of bio-preservation also preserves the original texture of the food [20,23]. Bio-preservatives (natural antimicrobial agents) can be divided into the following six groups; bacto-antimicrobials, phyto-antimicrobials, ovo-antimicrobials, milieu-antimicrobials, lacto-antimicrobials, and acid-antimicrobials. Lysozyme belongs to the ovo-antimicrobial group [1] and is widely used as a bio-preservative in the food industry [10,24]. Due to its high production rate, purity, higher potential against microbes and lower cost, it has a great importance in the industry. It is estimated that more than 100 tons of lysozyme are used industrially per year worldwide [25,26,27].

Among all bacteria, the genus *Bacillus* is considered the most suitable for the mass production of lysozyme [28,29,30,31], while among all *Bacillus* spp., *Bacillus subtilis* is the most popular species for higher lysozyme production. This is due to its excellent fermentation properties and tremendous ability to secrete proteins (lysozyme) directly into the growth medium, which offers great advantages in downstream processing, and it is nonpathogenic and free of endotoxins [29,32,33].

This study is an extension of our previous study. In the previous study, the lysozyme-producing probiotic bacterial species (*Bacillus*) were isolated from the soil samples. Following fermentation, various parameters were applied to the *Bacillus* species to determine the maximum lysozyme production. It was found that *Bacillus subtilis* BSN314 was able to achieve the highest lysozyme production [34]. In the present study, the lysozyme obtained from *Bacillus subtilis* BSN314 was purified by salt (ammonium sulfate) precipitation and dialysis methods and characterized by the bactericidal activity of lysozyme. The disintegrating effect of the purified lysozyme was investigated by atomic force microscopy (AFM). The pure form of the lysozyme was isolated, leading to the following three hypotheses for our further studies: (1) Following purification, the enzymatic activity of BSN314 lysozyme should remain higher. (2) The purified lysozyme should exhibit bactericidal action against different pathogenic bacterial strains. (3) Some specific peptides should be present in the purified lysozyme, which are responsible for the enzymatic and bactericidal activities. To evaluate these specific peptides, the extra purified lysozyme was subjected to a liquid chromatography-mass spectrometry (LC-MS) analysis. This study shows the significant importance of the extra-purified lysozyme isolated from *Bacillus subtilis* BSN314, which could be used in the pharmaceutical and food industries.

## 2. Results

### 2.1. Precipitation of Protein

**Figure 1:**
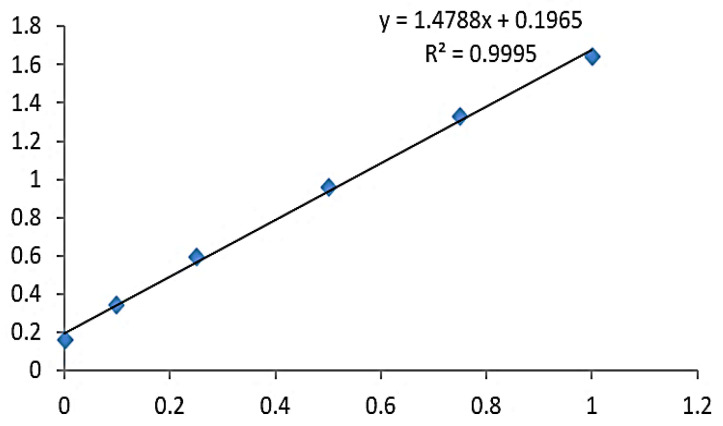
The standard curve for measuring the protein concentration (quantification) by the BCA (Bicinchoninic acid) method. *X*-axis; showing the concentration of protein mg/mL, *Y*-axis; showing the absorbance at 562 nm.

In the present study, lysozyme was purified at different ammonium sulfate concentrations. At each concentration of (NH_4_)_2_SO_4_, the amount of purified lysozyme and enzymatic activity were measured (Table 1). At an ammonium sulfate concentration of 70%, the protein/lysozyme concentration was highest (0.712 mg/mL), and the enzymatic activity was also at its maximum (43 U/mL). At a 20% ammonium sulfate concentration, the protein concentration was 0.24 mg/mL and enzyme activity was 14.3 U/mL, while at a 90% ammonium sulfate concentration, the protein concentration increased slightly (0.251 mg/mL), but the enzyme activity was lower (10.5 U/mL). The lysozyme concentrations obtained by different methods (ammonium sulfate precipitation, dialysis, ultrafiltration, and gel column chromatography) with total enzymatic activity, specific activity, and percent yield are shown in Table 2. The standard curve for measuring the protein concentration (quantification) by the bicinchoninic acid (BCA) method is shown in Figure 1. Using the standard curve, the protein concentration was calculated to be 0.099 mg/mL. The specific activity was calculated to be 221.52 U/mg and the yield was 8.55% (Table 2). 

The isolation and purification of lysozyme production by *Bacillus subtillis* BSN314 in different steps are listed in Table 3. The lysozyme produced by *Bacillus subtillis* BSN314 isolated by three sequential steps; ammonium sulfate precipitation, dialysis, and ultrafiltration. Following ultrafiltration, the resultant product was further used for the antimicrobial activity and AFM analysis. 

### 2.2. Antibacterial Activity of Lysozyme

**Figure 2:**
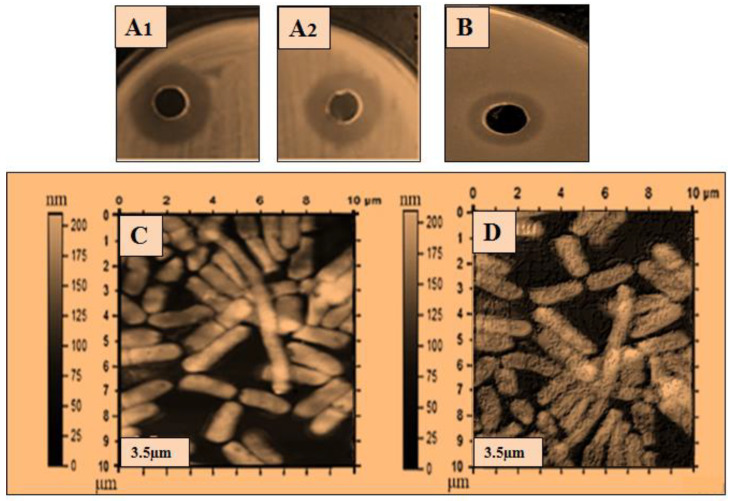
Antibacterial activity of lysozyme zone of inhibition (ZOI) (**A**,**B**): ZOI (**A1**,**A2**); of *Bacillus subtilis 168* by lysozyme, (**B**); ZOI of antibiotic, and AFM images: (**C**,**D**): (**C**); the structure of *Bacillus cereus*, before the application of lysozyme, (**D**); after the application of lysozyme representing the disintegrating effect of lysozyme on the bacterial surface.

**Figure 3:**
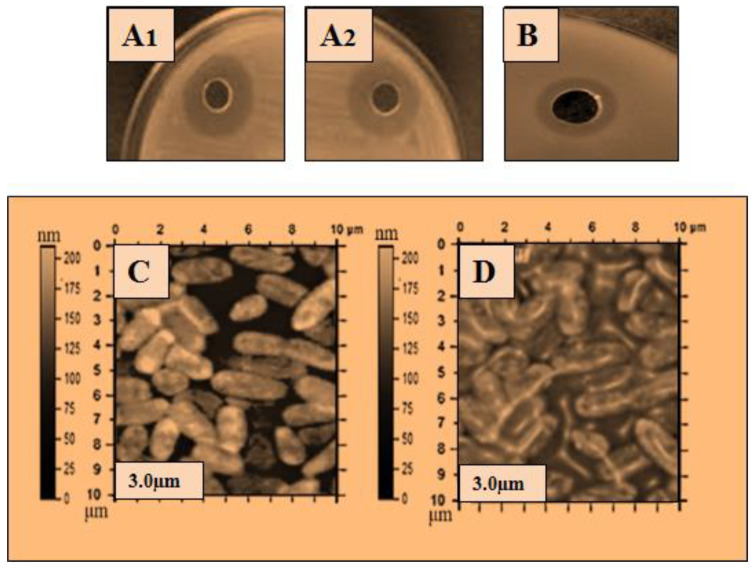
Antibacterial activity of lysozyme zone of inhibition (ZOI) (**A**,**B**): ZOI (**A1**,**A2**); of *Bacillus cereus* by lysozyme (**B**); ZOI of antibiotic, and AFM images: (**C**,**D**): (**C**); the structure of *Bacillus cereus*, before the application of lysozyme, (**D**); after the application of lysozyme representing the disintegrating effect of lysozyme on the bacterial surface.

**Figure 4:**
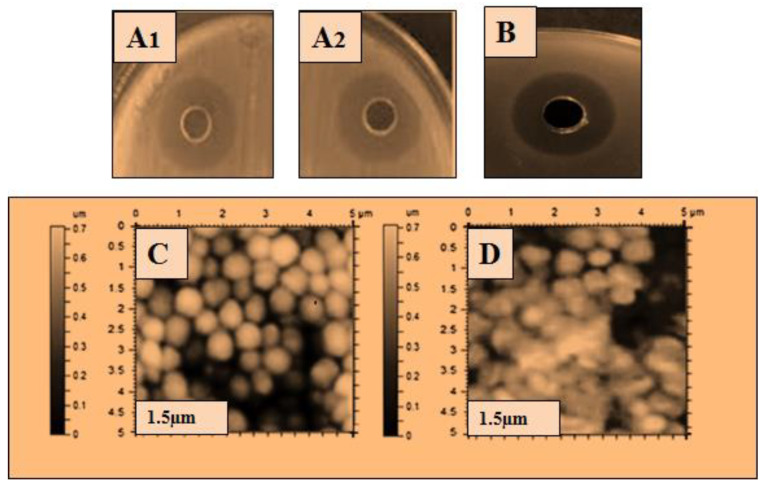
Antibacterial activity of lysozyme zone of inhibition (ZOI) (**A**,**B**): ZOI (**A1**,**A2**); of *Micrococcus luteus* by lysozyme (**B**); ZOI of antibiotic, and AFM images: (**C**,**D**): (**C**); the structure of *Micrococcus luteus*, before the application of lysozyme, (**D**); after the application of lysozyme representing the disintegrating effect of lysozyme on the bacterial surface.

**Figure 5:**
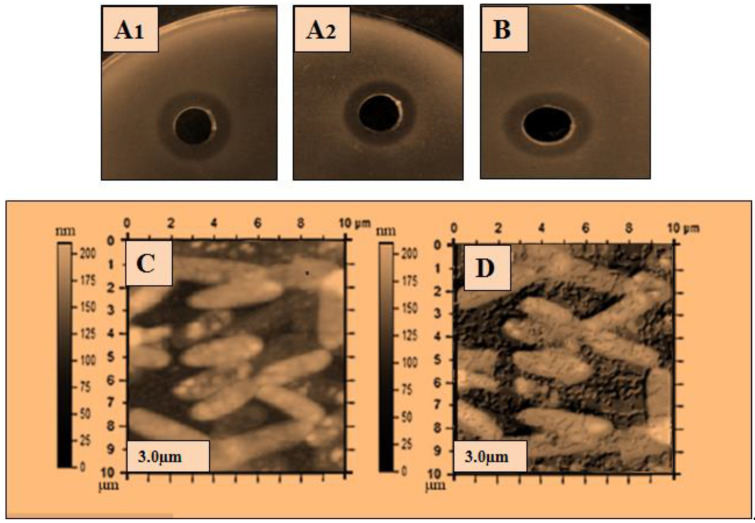
Antibacterial activity of lysozyme zone of inhibition (ZOI) (**A**,**B**): ZOI (**A1**,**A2**); of *Pseudomonas aeruginosa* by lysozyme (**B**); ZOI of antibiotic, and AFM images: (**C**,**D**): (**C**); the structure of *Pseudomonas aeruginosa*, before the application of lysozyme, (**D**); after the application of lysozyme representing the disintegrating effect of lysozyme on the bacterial surface.

**Figure 6:**
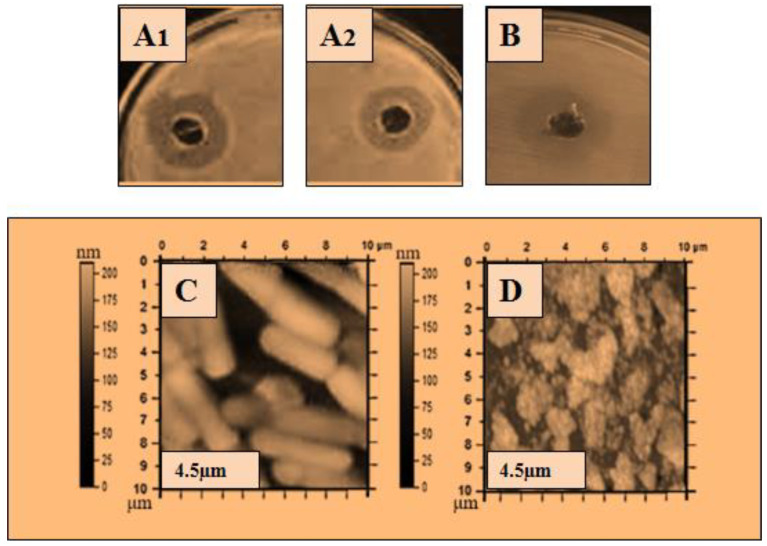
Antibacterial activity of lysozyme zone of inhibition (ZOI) (**A**,**B**): ZOI (**A1**,**A2**); of *Salmonella typhimurium* by lysozyme (**B**); ZOI of antibiotic, and AFM images: (**C**,**D**): (**C**); the structure of *Salmonella typhimurium*, before the application of lysozyme, (**D**); after the application of lysozyme representing the disintegrating effect of lysozyme on the bacterial surface.

The ultra-purified lysozyme was used for a further antibacterial assay, and atomic force microscopy (AFM) was used to determine the bactericidal action of the lysozyme on the bacterial cell. The purified lysozyme sample was subjected to antimicrobial activity against five bacteria: *Bacillus subtilis* 168, *Micrococcus luteus*, *Bacillus cereus*, *Salmonella typhimurium,* and *Pseudomonas aeruginosa*. The antibacterial results are shown in Table 3, and the zone of inhibition with the disintegrating effect of lysozyme on the bacterial cells is shown in Figure 2, Figure 3, Figure 4, Figure 5 and Figure 6. The results showed that the purified lysozyme obtained from *Bacillus subtilis* BSN314 exhibited a stronger antibacterial activity against the selected gram-positive strains, rather than the gram-negative bacterial strains. The results of the zones of inhibition (ZOI mm) and minimum inhibitory concentration/minimum bactericidal concentration (MIC/MBC μg/mL) against both the G-positive and G-negative bacterial strains are shown in Table 4. The strongest antimicrobial activity was observed against *Micrococcus luteus* (17 mm ZOI), while the lowest activity was measured against both gram-negative species, i.e., *Pseudomonas aeruginosa* and *Salmonella typhimurium* (12 mm). The minimum MIC/MBC was found to be 1.25 μg/mL against *Micrococcus luteus*, while the maximum MIC was measured against *Salmonella typhimurium* (2.75 μg /mL). To compare the antibacterial activity with the antibiotic (ciprofloxacin), two indices were used: antimicrobial index (AMI) and percent activity index (PAI). It was found that the purified lysozyme had a higher antibacterial activity against *Bacillus cereus* (AMI/PAI; 1.01/101) and *Bacillus subtilis 168* (AMI/PAI; 1.03/103), as compared to the antibiotic (ciprofloxacin) used in this study.

To observe the comparative effect of the purified lysozyme on the bacterial strains, the atomic force microscopy (AFM) technique was used. The disintegrating effect of lysozyme on the cells of the bacterial strains is shown in Figure 2C, Figure 3C, Figure 4C, Figure 5C and Figure 6C. The same concentration of lysozyme used for MIC and MBC was also used for the AFM analysis and showed the disintegrating effect on the bacterial cells (Figure 2C, Figure 3C, Figure 4C, Figure 5C and Figure 6C).

### 2.3. Assessment for the Composition of Lysozyme

For the evaluation of the different peptides found in the lysozymes, further assessments continued. For this purpose, the resulting protein solution was purified by gel column chromatography. The eluate was collected and its enzyme activity was assessed as 21.93 U. 

#### 2.3.1. Native-PAGE and Enzymatic Activities 

Following the gel column chromatography, the lysozyme was further processed by a native-PAGE analysis, and by this technique, the protein/lysozyme was obtained, whose enzymatic/cell lytic activity was determined to be 40.9 U/mL. This shows that the obtained protein was not denatured at this stage of purification, and the protein (lysozyme) still has cell lytic properties. Following the determination of the cell lytic activity of the purified lysozyme, the next step was to assess the composition of the lysozyme using various techniques, e.g., SDS-PAGE and LC-MS. 

#### 2.3.2. SDS-PAGE (Sodium Dodecyl Sulfate–Polyacrylamide Gel Electrophoresis) 

**Figure 7:**
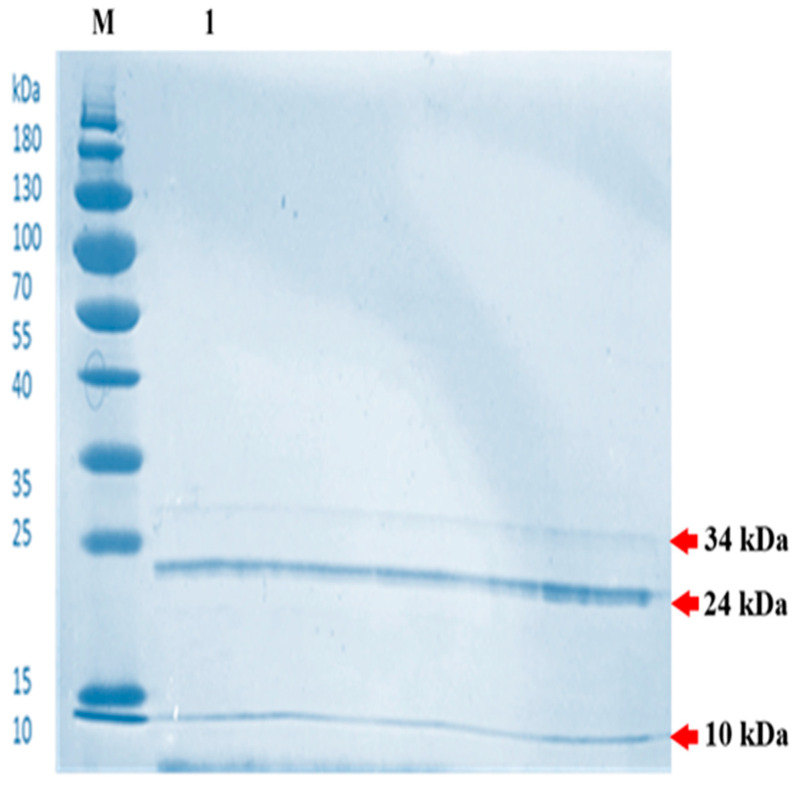
Analysis of *Bacillus subtilis* BSN314 lysozyme by sodium dodecyl sulfate-polyacrylamide gel (SDS-PAGE); Separation via SDS-PAGE resolves the purified protein into three distinct bands corresponding to molecular masses of 34 kDa, 24 kDa, and 10 kDa. This banding profile confirms the successful extraction and purification of the lysozyme to its fundamental monomeric form. Lane M contains the standard protein molecular weight marker; lane 1 contains the purified BSN314 lysozyme sample.

Following the gel column chromatography, the lysozyme was also analyzed by the SDS-PAGE technique. The SDS-PAGE analysis revealed three protein bands, indicating that the BSN314 lysozyme was purified to a monomeric protein (Figure 7). The resulting bands were observed at 34 kDa, 24 kDa, and 10 kDa, respectively. The exact molecular mass and purity of BSN314 lysozyme was determined by liquid chromatography-mass spectrometry (LC-MS). 

#### 2.3.3. Liquid Chromatography-Mass Spectrometry (LC-MS)

**Figure 8:**
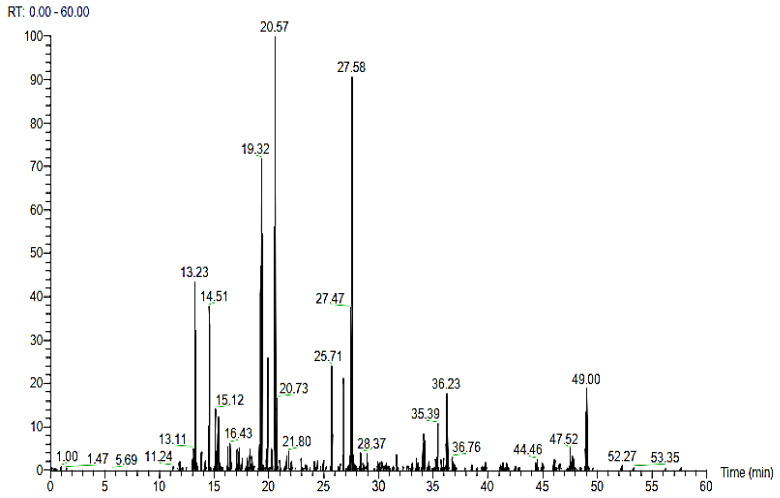
LC–MS (liquid chromatography–mass spectrometry) chromatogram, showing the detected protein components.

The bands of BSN314 lysozyme obtained by the SDS-PAGE analysis were cut-off, precipitated with acetic acid, enzymatically digested, desalted, and then analyzed by LC-MS. The chromatogram is shown in Figure 8. The LC-MS peak data showed that the number of proteins was 56, the number of peptides in the sample was 124, and the number of PSMs (peptide spectrum matches) was 309. The protein components found are listed in Table 5. Following the comparison with the PSMs database, only two components with molecular masses of 31.2 kDa and 48.6 kDa were found to be particularly relevant. These two peptides were A0A1Q9G213 (N-acetylmuramoyl-L-alanine amidase) and A0A1Q9FRD3 (D-alanyl-D-alanine carboxypeptidase). The nucleic acid sequence of these peptides was also obtained. 

#### 2.3.4. A0A1Q9G213: Protein Sequence

MVNIIQDFIP VGANNRPGYAMTPLYITVHNTANTAVGADA AAHASYLKNP DTATSWHFTVDDTEIYQHLPLNENGWHAGDGNGSGNRASIGIEICENADGDFAQAANAQLIKTLMSEHNISLANVVPHKYWSGKECPRKLLDTWDSFKAGIGGGGSQTYVVKQGDTLTSIARAFGVTVAELQEWNNIEDPNLIQVGQVLIVSAPSYAEEPELYPLPDGIIQLTTPYTSGEHVFQVQRALAALYFYPDKGAVNNGIDGIYGPKTADAVARFQSVNGLTADGIYGPTTKAKIAAQLS. 

#### 2.3.5. A0A1Q9FRD3: Protein Sequence

MNIKKCKQLLMSLVVLTLAVTCLAPMSKAKAASDPIDINASAAIMIEASSGKILYSKNADKRLPIASMTKMMTEYLLLEAIDQGKVKWDQTYTPDDYVYEISQDNSLSNVPLRKDGKYTVKELYQATAIYSANAAAIAISEIVAGSETKFVEKMNAKAKELGLTDYKFVNATGLENKDLHGHQPEGTSVSEESEVSAKDMAILADHLITDYPEILETSSIAKTKFREGTDDEMDMPNWNFMLKGLVSEYKKATVDGLKTGSTDSAGSCFTGTAELNGMRVITVVLNAKGNLHTGRFDETKKMFDYAFDNFSMKEIYAEGDQVKGHKTISVDKGKEKEVGVVTNKAFSLPVKNGEEKNYKAKVTLNKDTLTAPVKKGTKVGKLTAEYKGDEKDYGFLNSNLAGVDLVTKENVEKANWFVLTMRSIGGFFAGIWGSIVDTVTGWF.

## 3. Discussion

Currently, *B. subtilis* is of great interest to the secretome because it has a powerful machinery to produce proteins that are secreted from the cell and are free of toxins [35,36,37]. Researchers are trying to find techniques/methods to purify proteins secreted/produced by *B. subtilis* without denaturation. In this study, a multi-step methodology was used to purify the lysozyme product (protein) biosynthesized from the previously isolated strain *Bacillus subtilis* BSN314 [34]. This multistep methodology included ammonium sulfate precipitation, dialysis, ultrafiltration, and gel column chromatography. To obtain a maximum and highly purified lysozyme product, a similar multistep method has already been reported [2,38]. A high salt concentration reduces the solubility of the protein and also leads to protein denaturation. The main principle is that the high salt concentration combines with the water molecules on the surface of the protein and forms ions. These ions destroy the structure of the hydration membrane and make it denatured. These ions also lead to a decrease in the solubility of the protein [39]. However, some salts do not lead to protein denaturation, but these salts (in different concentrations) can be used to purify proteins from solutions by the process of precipitation [40]. Therefore, an appropriate salt concentration is necessary for the purification of protease. In the present study, the recovery rate of lysozyme was higher due to the salt (ammonium sulfate) precipitation method. The higher recovery rate could be explained by the salting-in and salting-out system. When (NH_4_)_2_SO_4_ was added to the aqueous protein solution at a low concentration, the solubility of the proteins in the solution gradually increased due to the ion concentration of the ammonium sulfate, which is called the salting-in effect. As the addition of (NH_4_)_2_SO_4_ increases, the solubility of the proteins begins to decrease, resulting in protein precipitation. Now, more protein is precipitated from the solution while less protein is soluble. This is called the salting-out effect [40,41]. The mechanism behind this system of salting-in and salting-out is explained by the hydrophobic interactions between proteins and water molecules. When ammonium sulfate is added to the protein solution, the surface tension of the solution (water plus protein) also increases. The increase in surface tension could also increase the hydrophobic interactions between protein and water molecules. As a result, the protein molecules begin to decrease their association with the solvent, so that the contact between the solvent and the protein decreases; at this stage, the protein begins to precipitate out of the solution [42]. 

Lysozyme produced by *Bacillus subtilis* BSN314 was purified in three sequential steps: sulfate precipitation, dialysis, and ultrafiltration. To determine the bactericidal potential of the purified lysozyme, the purified protein/lysozyme was used for bactericidal activity against some pathogenic bacteria (*Bacillus subtilis* 168, *Bacillus cereus*, *Micrococcus luteus*, *Pseudomonas aeruginosa*, and *Salmonella Typhimurium*). The results of ZOI and MIC/MBC showed that lysozyme had a higher antibacterial activity against gram-positive (*Bacillus subtilis* 168, *Bacillus cereus*, *Micrococcus luteus*), rather than gram-negative bacteria (*Pseudomonas aeruginosa* and *Salmonella Typhimurium*). In the previous literature, the same kind of results were recorded for the bactericidal activity of lysozyme [43,44,45,46]. The reason that gram-positive bacteria become more susceptible when lysozyme is applied, can be explained by the composition of the bacterial cell. Gram-positive bacteria have a thick peptidoglycan layer and no outer lipid membrane, whereas gram-negative bacteria have a thin peptidoglycan layer and an outer lipid membrane. The lipid layer provides maximum protection to gram-negative bacteria; therefore, gram-positive bacteria are easily killed by lysozyme [11,12,13]. A morphological examination of bacteria is considered important for investigating the bactericidal effect of an antimicrobial agent [47]. Atomic force microscopy (AFM) was used to study the morphological changes of the bacteria before and after the application of lysozyme on the bacterial cells. The cell wall was indeed damaged as the stiffness of the cell wall was reduced after treatment with lysozyme. The results of this study showed that after the application of lysozyme to the bacterial cells, deterioration occurred in both gram-positive and gram-negative species. Gram-positive bacteria were disintegrated at a low concentration of lysozyme, while a higher concentration was used for gram-negative bacteria. The lysozyme concentration used for the disruption of bacterial cells was the same as that used for MIC/MBC. Once cells are exposed to purified lysozyme, leakage and polarization of the cytoplasmic contents may occur, leading to cell disintegration. This disintegrating effect leads to the interruption of various vital functions of the cell and eventually to the death of the bacterial cells. Following the application of lysozyme to the cells, the AFM technique was used in the present study, and the final AFM images show the condition of the bacterial cells. Some of them were damaged while others were somewhat blurred. The same type of observation was recorded by Rao et al., (2017) [48]. The bacteriostatic effect of lysozyme is due to its ability to hydrolyze the β-l,4-glycosidic bond of peptidoglycans, which leads to the degradation of the murein (peptidoglycan) layer. As a result, the mechanical strength of the bacterial cell wall decreases, leading to the death of the bacterium [49,50,51].

The resulting purified protein (lysozyme) was further processed by gel column chromatography and then further processed to isolate the un-denatured protein by native-PAGE analysis. The purified/isolated lysozyme was found to still have cell lytic/enzymatic activity. To separate the expected peptides responsible for the biological activities (cell lytic and antibacterial activity), the SDS-PAGE technique was used (to separate the peptides with masses between 5 and 250 kDa). The sequences of the primary protein standards were monitored by SDS-PAGE [52]. Following the isolation of the enzymatic peptides from the SDS-PAGE analysis, the resulting bands were 34 kDa, 24 kDa, and 10 kDa, respectively, indicating that the peptides isolated from BSN314 lysozyme were in the form of a monomeric protein (due to low molecular weight). A similar result was obtained by Chen et al., (2019) [38]. The exact molecular mass and chemical composition of the isolated peptides were determined by an LC-MS analysis. Liquid chromatography-mass spectrometry is now an established technique for protein analysis that can provide both qualitative and quantitative information about the proteins of biological samples [53]. The LC-MS peak data showed that the total number of proteins was 56, the total number of peptides in the sample was 124, and the number of PSMs (peptide spectrum matches) was 309. 

The PSM data showed that among the 309 matched peptide spectra, only two components with molecular masses 31.2 kDa and 48.6 kDa, were considered particularly relevant. These two components were; A0A1Q9G213 (N-acetylmuramoyl-L-alanine amidase) and A0A1Q9FRD3 (D-alanyl-D-alanine carboxypeptidase). They may be responsible for antibacterial and enzymatic activities [6,54,55,56]. These two peptides (A0A1Q9G213 and A0A1Q9FRD3) of *Bacillus subtilis* BSN314 lysozyme might be responsible for the antibacterial and enzymatic activities. They prevent the growth of bacteria. The results of antibacterial activity, the AFM analysis, and cell lytic activity indicated that BSN314 lysozyme has a strong potential against bacteria and can disintegrate the bacterial cell. The same type of observation was made in previous studies [57,58,59]. Possibly, N-acetylmuramoyl-L-alanine amidase and D-alanyl-D-alanine carboxypeptidase could be responsible for the bactericidal effect. Previous studies indicated that N-acetylmuramoyl-L-alanine amidase and D-alanyl-D-alanine carboxypeptidase have antibacterial and cell lytic activities [60,61,62,63,64,65]. 

## 4. Materials and Methods

### 4.1. Reagents and Chemicals

Analytical grade sodium chloride (≥99.5%), sodium dihydrogen phosphate dodecahydrate (≥99.0%), disodium phosphate (≥99.0%), sodium hydroxide (≥98.0%), ammonium sulphate (≥99.0%), ethanol, 5× protein loading buffer, and *glacial acetic acid* were purchase from Beijing Chemical Plant. While yeast extract PL0021 (biological grade) and soy peptone (biological grade) were purchased from Beijing Aoboxing Biotechnology Co. Ltd. (Beijing, China). 

Agar (biological grade) was provided from CHEMBASE Company (Nantong, China); 50 × TAE, Tris-HCl, glycine, SDS, Coomassie brilliant blue, IPTG, kanamycin, agarose gel electrophoresis, and surfactant Tween 20 and Tween 80 were purchased from Biotopped Technology Co. Ltd (Shanghai, China). Analytical pure 5 × non-denatured protein loading buffer, 30% acrylamide, and 10% ammonium persulfate were purchased from Fujian Ming Lin Technology Co. Ltd (Longyan, China). While tetramethylethylene diamine (TEMED) ≥ 99%, 1.5 M Tris-Hcl (pH 8.8), 1.0M Tris-Hcl (6.8), non-denatured protein running buffer, a BCA protein assay kit, were provided by Biyuntian Co.(Shanghai, China).

Protein marker was purchased from Thermo Fisher Scientific (Waltham, MA, USA). SDS-PAGE gel preparation kit, DNA Marker DL 2000, and DL 5000 were purchased from Beijing Zhuangmeng International Biogenomics Division Technology Co. Ltd. (Beijing, China). Nucleic acid dyes T4 was obtained from TAKARA Company (Toshima-ku, Japan) and DNA ligase, BamHI restriction enzyme, XhoI restriction enzyme, and DNA agarose gel recovery kit from OMEGA company (La Chaux-de-Fonds, Switzerland).

TIANamp Bacteria DNA Kit (DP302) was purchased from Tiangen Biotech. (Beijing) Co. Ltd., while analytical grade agarose was obtained from Biowest (Nuaillé, France), and SDS-PAGE kit from Beijing Zhuangmeng International Biogenomics Division Technology Co. Ltd. Glucose anhydrous (≥99.0%), glycerin, and hydrogen peroxide (≥35.0%) were purchased from Sinopharm Chemical Reagent Co. Ltd. (Shanghai, China), while Ex Red and DL2000 plus were purchased from Beijing Zhuangmeng Bio. PCR pre-mix (Prime STA R HS) was purchased from Takara Bio Company (Toshima-ku, Japan).

### 4.2. Equipment Used

An analytical balance (model No. BSA 224S), manufactured by Sartorius Scientific Instruments (Beijing, China) Co., Ltd.; an ultra clean work table, type SW-CJ-1FD, made by Sujing Group Suzhou Antai Air Technology Co., Ltd.; a high-pressure steam autoclave (model No. HVA-100), made by Hirayama (Minato, Japan); a pH meter (model No. FE20) equipped with a Lab Pure Pro-ISM probe, made by Mettler instruments Shanghai Co., Ltd.; a visible spectrophotometer (model No. 722), made by Shanghai Jinghua Technology Instrument Co., Ltd.; a mini spin centrifuge (model Eppendorf AG 2231), made by Germany; a nucleic acid electrophoresis apparatus (model No. DYY-6C), produced by the Beijing Liuyi Instrument Factory; a gel imaging system (model No. UVP Bio Spectrum 510), supplied by Ultra-Violet Products Ltd. Cambridge, UK; a bench shaking incubator (model No. IFORS AG CH-4103), made in Bottmingen Switzerland; a thermal cycler type PCR (model No. S1000) instrument, made by Bio-Rad (Hercules, CA, USA), and a microwave digestion system (model No. 910980) were used for this study. 

Furthermore, a benchtop refrigerated centrifuge (model no CT15RE), made by HITACHI (Chiyoda City, Japan), a biochemical incubator (model Bluepard), manufactured by Shanghai Yiheng Scientific Instrument Co., Ltd.; a magnetic stirrer (model RCTBS025 IKA) and dialysis bag, manufactured by Beijing Boya Red Star Biotechnology Co. Ltd.; a protein purifier (model No 25), made by AKTA Pure company; an Infinite microplate reader (model No M200PRO) from Switzerland TEC company; a decoloring shake, made by Beijing Liuyi Instrument Factory; a protein electrophoresis system, by BIO-RAD (Hercules, CA, USA); an ice maker (model No FM40), produced by the snow ice making company, and an ultrasonic disruptor, manufactured by Galanz (Foshan, China), were also used in this study.

### 4.3. Common Solutions and Media Preparation

The modified medium (MM) ingredients were; 0.5% yeast extract, 2.5% soy peptone, 1.5% glucose, 0.5% K_2_HPO_4,_ and Tween-20 0.1%. For the solid medium: 1.8% (*w*/*v*) agar powder was added. While the LB liquid medium was prepared by adding 0.5% (*w*/*v*) yeast extract, 1% (*w*/*v*) peptone, and 1% (*w*/*v*) sodium chloride. All of the media were sterilized at 121 °C for 20 min. For the kanamycin solution, 50 mg/mL mother liquor was prepared, after that it was diluted with sterile distilled water until a 50 µL/mL concentration was obtained. 

A 5 × Tris-glycine buffer solution was made by mixing 15.1 g Tris, 94 g glycine, and 5 g SDS; it was then stirred with 1 L of ionized water and then stored at room temperature. A phosphate buffer (PB) was prepared by mixing of 1.217 g of sodium dihydrogen phosphate dihydrate, 4.370 g of disodium hydrogen phosphate dodecahydrate, and 1 L of deionized water, to obtain 20 mM phosphoric acid with pH 7.0 buffer. While the phosphate buffered saline (PBS) was formulated by adding 0.609 g sodium dihydrogen phosphate dihydrate, 2.185 g disodium hydrogen phosphate dodecahydrate, 29.220 g sodium chloride, and 500 mL deionized water, to obtain 20 mM phosphoric acid with pH 7.0. 

The Coomassie brilliant blue staining solution was prepared by adding 0.1 g Coomassie brilliant blue R-250, 25 mL of isopropanol, and 10 mL of glacial acetic acid. Then, it was stirred well, to make a constant volume of 100 mL, while the Coomassie brilliant blue staining decolorizing solution was made with 100 mL acetic acid and 50 mL ethanol. Then, 850 mL deionized water was added and the solution was then stored at room temperature. Then, 24.2 g Tris, 5.7 mL glacial acetic acid, and 10 mL 0.5 mol EDTA, were added to the prepared agarose gel electrophoresis buffer, and deionized water was added to make a volume of 100 mL. 

### 4.4. Strain Activation and Inoculum Preparation

The previously isolated strain *Bacillus subtilis* BSN314 (which was able to produce the higher rate of lysozyme) was used for the isolation and purification of lysozyme, and *Micrococcus luteus* was used for the cell lytic/enzymatic activity of the purified lysozyme. These two strains were stored at −80 °C. For activation, the culture was taken, spread on Luria agar (LA) medium and allowed to grow at 37 °C for 24 h. A single colony was selected for further inoculation in 4 mL of Luria broth (LB) medium in a 10 mL shaking tube, and incubated for 24 h at 37 °C and at 220 rpm. To obtain a second generation of cultures, 1 mL of the inoculum was transferred to 100 mL of the LB medium and incubated for another 24 h under the same growth conditions [34]. 

### 4.5. Isolation and Purification of the Lysozyme Product

Initially, 1 mL of *Bacillus subtilis* BSN314 culture was transferred into a 250 mL sterilized flask which contained the 100 mL MOM medium (modified optimal medium), and incubated at 37 °C, 220 rpm for 24 h. The broth culture was centrifuged at 8000 rpm for 20 min to remove the bacteria, the supernatant was used for following steps. Ammonium sulfate precipitation, dialysis, ultrafiltration, and gel column chromatography methods were designed for this study to obtain the maximum product and high level of lysozyme produced by *Bacillus subtilis* BSN314. 

#### 4.5.1. Ammonium Sulfate Precipitation

Isolation of lysozyme was performed according to the method described by Chen et al., (2019) [38]. This method started with the slow addition of solid powdered ammonium sulfate to the fermented supernatant of *Bacillus subtilis* BSN314 (at 4 °C). A magnetic stirrer was used to increase the saturation of ammonium sulfate in the solution to 20%, 30%, 40%, 50%, 60%, 70%, 80%, 90%, and 100%. Then, the solution was centrifuged at 1000 rpm for 30 min under refrigerated conditions, and the precipitate was carefully collected and dissolved in phosphate buffer solution (PB). Protein concentration was determined by the bicinchoninic acid (BCA) method [66,67] and enzyme activity was determined by the turbidimetric method [2,68,69]. 

#### 4.5.2. Desalination by Dialysis

The protein solution contained ammonium sulfate, which was removed by the dialysis bag method [70]. A 15 cm dialysis bag was taken, boiled with 2% sodium bicarbonate and 1 mmol/L EDTA solution for 10 min, and then rinsed with deionized water. The dialysis bag was closed with a clip, and the precipitation solution obtained in the previous step was poured into the dialysis bag while the air clip was sealed at the other end. The bag was immersed in 2 L of deionized water, stirred slowly with a magnetic stirrer, and dialyzed for 24 h at 4 °C. The dialysis bag was changed every 6 h to improve and accelerate the dialysis rate. The dialysate was used as crude extract for further purification. Protein concentration was carefully determined by the bicinchoninic acid (BCA) method, and enzyme activity was determined by the turbidimetric method.

#### 4.5.3. Ultrafiltration (UF) Tube (10 KD)

In this method, the lysozyme product was concentrated using an ultrafiltration (UF) 10 KD tube (Utra-15; Millipore Amicon, Bedford, MA, USA) [3]. The UF 10 KD tube was first soaked with 0.2 mol/L sodium hydroxide for 20 min and then with deionized water for 6 h. Following the pretreatment, a small amount of deionized water was added to the UF 10 KD tube and centrifuged at 4000 rpm and 4 °C for 10 min. The supernatant was used for further experiment. The centrifugation was repeated until the solubilization of the protein reached a maximum. When the supernatant reached approximately 3 mL, this step was terminated. This ultra-purified product was used for a further antibacterial assay, and atomic force microscopy (AFM) analysis. For the determination of peptides and their chemical composition, the ultra-purified product was further processed by gel column chromatography. 

### 4.6. Gel Column Chromatography

For further purification, an AKTA Purifier 25 system (Amersham Bioscience, Piscataway, NJ, USA) was used to isolate lysozyme. The sample (10 KD) collected from the ultrafiltration (UF) tube was loaded into a HiTrap TM Desalting (GE Healthcare) gel column chromatography. The column was fully equilibrated with PB solution pH 7.0 to purify the lysozyme [71,72]. The flow rate was 0.1 mL/min for elution with the equilibrium solution, detection was performed at 280 nm, and the activity peaks were collected while the active frictions were collected in sterilized 10-mL tubes. The active fractions containing lysozyme were again concentrated by using an ultracentrifuge tube (Utra-15; Millipore Amicon, Bedford, MA, USA) centrifuge at 4000 r/min at 4 °C for 10 min. Once the active concentrated fraction of lysozyme was obtained, the following steps were conducted to determine the total protein quantification (concentration) the by BCA (Bicinchoninic acid) method [66,67] and the enzyme activity by the turbidimetric method [2,68,69]. The purified sample obtained from this step was further assessed by polyacrylamide gel electrophoresis (PAGE). 

### 4.7. Quantification of the Total Protein

The total protein content from the purification was measured using the modified colorimetric method [66,67] using a bicinchoninic acid (BCA) protein assay kit (Thermo Scientific™ Pierce™ Catalog number: 23225). Briefly described, the protein standards were prepared by completely dissolving the protein standard, taking a 10 μL standard, and diluting it in 100 μL PBS to reach the final concentration of 0.5 mg/mL. The diluted 0.5 mg/mL protein standard was stored at −20 °C. The BCA working solution was prepared at a ratio of 50:1, i.e., 50 volumes of BCA reagent A plus 1 volume of BCA reagent B (50:1), mixed thoroughly, and the BCA working solution was stable for 24 h at room temperature. Then, 0, 0.1, 0.25, 0.5, and 0.75 mg/mL standard solutions were added in 96-well plates, 20 μL of each sample was added, then 200 μL of the BCA working solution was added to each well and stored at 37 °C for 30 min. Absorbance was measured at 562 nm using a microplate reader. The protein concentration was calculated using the standard curve.

### 4.8. Polyacrylamide Gel Electrophoresis (PAGE)

This is a technique used to separate proteins, based on their shape, size, mass, and charge. There are two types: native PAGE and sodium dodecyl sulfate (SDS) PAGE. 

#### 4.8.1. Native-PAGE (Native Polyacrylamide Gel Electrophoresis)

Native-PAGE is an electrophoretic technique that separates proteins, based on their size and charge. A gel kit purchased from Beijing Zhuangmeng International Bio Genomics Division Technology Co. was used for this analysis. Preparation of the native-PAGE gel began with making a 12% separation glue to which 10% ammonium persulfate was added and immediately poured in the glue. We left a height of about 1.5 cm for sample loading. Then, 75% ethanol was poured between the two plates to remove air and promote the solidification of the separating gel. We prepared a 5% concentrated adhesive, added the appropriate 10% ammonium persulfate, and mixed well. Then, we poured the adhesive and immediately inserted the comb. Prior to loading the samples into the gel hole, it was evenly mixed with 5× non-denatured protein loading buffer, and electrophoresis was performed at a constant voltage of 100 V. The protein obtained from the native-PAGE can be used for the determination of cell lytic/enzymatic activity [2]. 

#### 4.8.2. SDS-PAGE (Sodium Dodecyl Sulfate–Polyacrylamide Gel Electrophoresis)

SDS-PAGE is a separation technique that separates proteins on the basis of their mass. SDS-PAGE is used to monitor the purification process and to determine the homogeneity and apparent molecular mass of the purified lysozyme. SDS–PAGE was performed on an electrophoresis apparatus (Bio-Rad Laboratories, Hercules, CA, USA), with a 12% (*w*/*v*) acrylamide separating gel and a 5% (*w*/*v*) stacking Quick SDS-PAGE Gel Preparation Kit, according Laemmli’s method [24]. SDS-PAGE was performed by electrophoresis, at a constant voltage of 100 V, when the bands entered the separation gel, we adjusted the voltage to 120 V for electrophoresis. Following the run, the protein bands in the gel were stained with Coomassie brilliant blue dye R-250 for 30 min, the SDS-PAGE electrophoresis was over, we used the gel imaging system to photograph the gel and cut the corresponding bands for further liquid chromatography-mass spectrometry analysis [73].

### 4.9. Liquid Chromatography-Mass Spectrometry (LC-MS)

#### 4.9.1. Sample Preparation

BSN314 lysozyme samples were processed and enzymatically digested by the SDS-PAGE gel. Using a scalpel blade to cut the target bands, we cut 3 small pieces, 1 mm in size, and transferred them in a 1.5 mL EP tube 600 μL. Decolorizing solution was added to each tube to decolorize the sample, the samples were washed until transparent, and then we removed the supernatant. Then, 300 μL of 100% ACN ThermoMixer was added, shaken for 5 min until the gel particles turned white, we sucked off the ACN, and freeze-dried the samples for 3 min. For the protein reduction, 300 μL of 10 mM DTT/50 mM NH_4_HCO_3_ was added to each 1.5 mL tube. Then, they were shaken well and incubated for 1 h in a 56 °C chamber (500 mM dithiothreitol (DTT) = 0.077 g/mL), then we discarded the supernatant.

The samples were dried by adding 300 μL of 100% ACN ThermoMixer and shaken for 5 min until the glue particles turned white, we sucked off the ACN, and freeze-dried the samples for 3 min. Then, we added 300 μL 60 mM IAA/50 m NH_4_HCO_3_ to each tube. Each tube was shaken and the samples were mixed well and then incubate for 30 min in the dark until the gel block became swollen and transparent (600 mM iodoacetamide (IAA) = 0.111 g/mL). Once the gel particles were again dried by adding 300 μL of 100% ACN Thermo Mixer, and shaken for 5 min until the rubber particles turned white, we sucked off the ACN, and freeze-dried the samples for 3 min. Then, 50–80 μL of 50 mM ammonium bicarbonate solution was transferred to each tube, then 1–2 μg of pancreatin was added, and we squeezed the gel with the help of a glass rod. Prior to digestion, the sample was incubated at 37 °C for more than 6 h. 

#### 4.9.2. Digested

The samples were digested with trypsin, which was re-suspended in 10 mM acetic acid, the recommended concentration is 0.25 μg/μL, and the optimal pH of trypsin was 7–8.5. Therefore, after adding trypsin, 1 μL of protein solution was taken to measure the pH. Then, 200 μL of acetonitrile containing 0.1% formic acid (FA) was transferred to each EP tube, shaken for 5 min, and the supernatant was transferred to a clean 1.5 mL EP tube. Then, 30 μL of 0.1% FA was added into the gel, shaken it for 5 min, and 200 μL of 0.1% FA containing acetonitrile was added and the solution was shaken for 5 min. The supernatant was aspirated and both supernatants were combined in a 1.5 mL EP tube. The sample was concentrated by using a centrifugal concentrator at room temperature. 

#### 4.9.3. Desalination

Desalination of the sample was carried out using C_18_-resin (Thermo Fisher Pierce™ C18 Tips, 100 µL bed, Cat #: 87782) to obtain the concentration of, desalting, and obtaining the elution of the peptides for the LC-MS mass analysis [74]. Briefly described, the method was started by putting the C_18_- resin tip on the pipette, we wet the dry C_18_-resin by aspirating the wet solution containing 0.1% trifluoroacetic acid (TFA) in 50% acetonitrile and dispensing it to the waste. We repeated this 5 times. Then, we equilibrated the C_18_-resin for peptide binding by aspirating the equilibrium solution containing 0.1 TFA and dispensing it to the waste. We repeated this 5 times. The sample peptide was bound to the C_18_-resin by the repeated aspiration and dispensed in the same vial 10 times. Following the binding, next step was to wash away the salts from the protein peptide by aspirating the wash solution, containing 0.1% TFA and dispensing it to the waste. It was repeated 5 times to remove the maximum amount of salts. The elocution of the peptide was achieved by the 5 repetitions, then slowly aspired and dispensed in the same 180 µL elocution buffer vial, containing 0.1% formic acid. 

#### 4.9.4. Conditions for Liquid Chromatography–Mass Spectrometry (LC-MS)

The high performance liquid chromatography (HPLC) EASY-nLC™ 1200 combined with mass spectrometry (MS) was applied for the proteomic data acquisition. The digested samples (2 μg) were loaded to the column (C18, 2 μm, 75 μm × 250 mm) with the flow rate of 300 μL/min. The mobile phase A (0.1% formic acid in water) and B (0.1% formic acid in 80% acetonitrile), were used as gradients, as shown in Table 4. The eluent entered the mass spectrometry detector and the mass spectrometer, which were an Orbitrap mass spectrometer with a spray voltage system: 2.2 kV; the capillary temperature: 320 °C; the parent ion scan range: *m*/*z* 350–1500; the product ion scan range: started from *m*/*z* 120; the max IT: first level 20ms, second level 45 ms; the resolution was at: level 1 60,000@*m*/*z* 200, level 2 15,000@*m*/*z* 200; the data-dependent MS/MS: top 20; isolation window: 1.6 Da; collision energy: 27%. 

Protemo Discoverer 2.2 software was used for data processing and Uniprot was used for the analysis of the protein library. As a condition, trypsin was digested with a maximum of 2 missing sites; fixed modification: C (+57.021), variable modification M (+15.995) N (+42.011); the mass deviation for MS1 was 10 ppm, MS2 was 0.02 Da; FDR 1%. 

### 4.10. Antibacterial Assay

The antimicrobial activity was determined by using the well diffusion method. The supernatant obtained from the ultrafiltration method (pure lysozyme), was used for the antibacterial assay. Using the freeze-drying method, the supernatant was obtained in the form of powdered metabolites, which were used for further antibacterial activity [75,76]. 

Around 3 gram-positive bacteria, including; *Bacillus subtilis 168*, *Bacillus cereus*, and *Micrococcus luteus,* and two gram-negative species, *Pseudomonas aeruginosa* and *Salmonella Typhimurium* were used. These bacterial strains were indigenously obtained from the clinical source. For the susceptibility tests, the standard solution of ciprofloxacin (10 μg/mL) was used. This drug was used as the positive control, deionized water was used as the negative control. The zones of inhibition (ZOI), produced by the lysozyme were compared with ZOI (mm) of the standard drug (positive control). 

The selective bacterial strain cultures were grown overnight in Mueller–Hinton agar (MHA) and Sabouraud dextrose broth (Oxoid, U.K), then centrifuged at 9000× *g* and the supernatant removed. A cell suspension equivalent to 0.5 McFarland standard (1.5 × 108 CFU/mL), was prepared in a 5.0 mL sterile physiological saline solution. Confluent lawns were made on fresh MHA and Sabouraud dextrose agar (SDA) plates and allowed to diffuse for 5–10 min. The wells were dug (6 mm), stock solutions of 10 μL purified lysozyme were dispensed into them and allowed to diffuse into the media for 15–20 min. The plates were incubated at 37 °C for 24–48 h [75,76]. For the reproducibility of the results, all of the experiments were repeated thrice.

#### 4.10.1. Minimum Inhibitory Concentration (MIC) and Minimum Bactericidal Concentration (MBC) Assays

A modified dilution method was used for the determination of MIC and MBC. Lysozyme was diluted at different concentrations (1, 1.25, 1.75, 2, 2.25, and 2.75 µg/mL) in sterile nutrient broth in test tubes. Using a sterile wire loop, a culture of each bacterial strain was inoculated into test tubes containing 1 mL of the various concentrations of lysozyme in the nutrient broth. The tubes were incubated at 37 °C for 24 h and growth was observed by turbidity. At the concentration where no growth/turbidity occurred, a sterile culture swab stick was dipped into the broth from each test tube and was loaned to the agar culture plates. Equal volumes of sterile nutrient broth were added to the test tubes containing broth media and incubated at 37 °C for 24 h (Supplementary Material). The tubes and agar plates were examined for growth or turbidity. These experiments were repeated three times [75,76,77].

#### 4.10.2. Antimicrobial Index (AMI)

The AMI was calculated by the following formula;

$$\mathrm{AMI}=\frac{\mathrm{ZOI}\;\mathrm{of}\;\mathrm{Purified}\;\mathrm{Lysozyme}}{\mathrm{ZOI}\;\mathrm{of}\;\mathrm{Antibiotic}}$$

AMI values > 1 indicating extract is more active than the standard drug (antibiotic) while the AMI < 1 indicating extracts are less active than the standard drug [75,76]

#### 4.10.3. Percentage Activity Index (PAI)

It is the ratio between the zone of inhibition by the pure lysozyme to the zone of inhibition of the antibiotic, and multiplied by 100.

$$\mathrm{PAI}=\frac{\mathrm{ZOI}\;\mathrm{of}\;\mathrm{Lysozyme}}{\mathrm{ZOI}\;\mathrm{of}\;\mathrm{Antibiotic}}\times 100$$

value > 100 showing that the extract is more potent than the antibiotic, while <100 shows that the extract is less potent than the antibiotic [76].

#### 4.10.4. Atomic Force Microscopy (AFM) Analysis

In the present research work, the gram-positive (*Bacillus subtilis 168, Bacillus cereus*, and *Micrococcus luteus)* and gram-negative *(Pseudomonas aeruginosa* and *Salmonella Typhimurium*) strains were used for the atomic force microscopy (AFM) analysis. The selected bacterial cells were collected from each of the activated stock cultures in an Eppendorf (1.5 mL) tube, centrifuged at 8000 rpm for 5 min, and the bacterial residues were collected and the supernatant discarded. The centrifugation process was repeated three times to obtain sufficient cells. The effective concentrations of purified lysozyme were prepared (same as used for MIC/MBC) in the deionized water, added to the isolated cells of each strain and allowed to stand in a shaking incubator (100 rpm) for 3 h. 

The atomic force microscopy (AFM) analysis was performed according to the method of Usmani et al., (2021) [78] with some modifications. The method was started by treated cells placed on a clean glass slide and a thin smear was made with the help of a wire loop. The slides were air dried under sterile conditions at 25% relative humidity. The AFM images were acquired using Nano-scope IIIA with the J-scanner in tapping mode. Silicon nitride cantilevers (Olympus AC240TS) with a curvature radius of 20 nm, a resonance frequency of 70–100 kHz, a scan rate of 1 Hz, and a stiffness of 5 N/m, were used to examine the samples (Figure 2, Figure 3, Figure 4, Figure 5 and Figure 6). 

### 4.11. Assay of Lysozyme Activity

The lysozyme assay or the cell lytic activity was assessed by the turbidimetric method [1,3,67,68]. During this method, *Micrococcus luteus* was used as substrate. It was cultured in a Luria broth (LB) medium in a shaker tube. The speed of the shaker tube was set at 200 rpm while it was incubated at 37 °C for 24 h. Following the incubation period (24 h), the culture was added to the centrifuged tubes and was centrifuged at 8000 rpm for 10 min. The residue and supernatant were obtained and the supernatant was discarded. The residue containing the pellets of *Micrococcus luteus* was poured into a phosphate buffer solution (pH 6.2). Then, 2 mL of this solution was added to a cuvette, and the optical density (OD) was adjusted to 0.8 (OD_450_).Then, 0.5 mL of the selected bacterial strain (sample) was taken from the fermentation broth culture and placed in the cuvette that already contained 2 mL of *Micrococcus luteus* and the PB suspension. Now, both were mixed well. Pure liquid Luria broth (LB) medium was used as a control. The optical density (OD) values were measured at 450 nm after the intervals of 15–75 s. All samples were repeated at least 5 times. The lytic activity of the enzyme (lysozyme) was calculated under the conditions of: 25 °C, pH 6.2, and at a wavelength of 450 nm. The difference between the two OD_450_ values (for the samples and control) was calculated as ∆A_450_. The calculation was carried out by the unit of activity corresponding to the amount of enzyme required to decrease the absorbance by 0.001 per minute. The enzyme activity was calculated by the following formula:

$$I=\frac{A1-\;A2}{0.001\times \;\mathrm{Ew}}$$

where I = enzyme activity, A1 = absorbance at 450 nm, A2 = absorbance after 1 min, A1 − A2 = the change in absorbance per minute at 450 nm, 0.001 = decrease the absorbance value at 450 nm by 0.001 per minute as an activity unit. Ew = 0.5 mL detection enzyme solution contains the mass (mg) or volume (mL) of the original enzyme solution [34]. 

## 5. Conclusions

Lysozyme is presented as a natural antimicrobial agent against pathogens. In the present study, the lysozyme was purified. The observations of the results showed that the purified lysozyme exhibited a high potential against some pathogenic bacteria. In this study, two components were identified, namely A0A1Q9G213 (N-acetylmuramoyl-L-alanine amidase) and A0A1Q9FRD3 (D-alanyl-D-alanine carboxypeptidase). It was concluded that these components may be responsible for enhancing the bactericidal activity of lysozyme. This purified lysozyme could be used to control microbial infections in the host by killing the microbes and strengthening the host’s defense mechanisms against microbial pathogens. This could contribute to the development of a strategy of biological control of bacterial infections in humans and animals. Further research is needed to identify the antibacterial mechanism of the purified components of lysozyme. Due to its strong bactericidal potential, the lysozyme purified from *Bacillus subtilis* BSN314 could play an important role in the food industry. It can be used as a dietary supplement to boost immunity against pathogens and could also be used as a natural preservative in the food industry. Future gene identification and expression studies may lead to improve the importance of lysozyme biosynthesized from *Bacillus subtilis* BSN314. 

## Figures and Tables

**Table 1:** Ammonium sulfate precipitation at different concentrations.

Ammonium Sulfate Conc.	Vol. (mL)	Protein Conc. (mg/mL)	Enzyme Activity (U/mL)
20%	6	0.24	14.3 ± 0.9
30%	6	0.125	0
40%	6	0.123	0
50%	6	0.131	0
60%	6	0.124	0
70%	6	0.712	43 ± 1.5
80%	6	0.203	12.2 ± 1.0
90%	6	0.251	10.5 ± 0.7
100%	6	0.373	15 ± 1.2

The enzyme unit (symbol U), is a unit of the enzyme’s catalytic activity. One U (μmol/min). As 1 mL was taken for the enzymatic activity, so unit is U/mL.

**Table 2:** The results of the total activity, protein concentration, specific activity, and yield% data of each step in the separation and purification processes (ammonium sulfate precipitation, dialysis ultrafiltration, and gel column chromatography) are shown.

Steps	Vol. mL	Total Enzyme Activity U	Protein Conc. mg/mL	Specific Activity U/mg	Yield %
Ammonium sulfate precipitation	6	258	0.712	362.36	100
Dialysis	20	193.5	0.422	458.53	75
Ultrafiltration	3	153	0.318	481.13	59
Gel column chromatography	2	21.93	0.099	221.52	8.55

**Table 3:** Antimicrobial activity indicating zone of inhibition (ZOI mm), minimum inhibitory and bactericidal concentrations (MICs and MBCs) of the purified lysozyme (produced by *Bacillus subtilis* BSN314) against the gram positive and gram negative bacteria. CIP indicating ciprofloxacin, with ZOI (mm). Antimicrobial index (AMI) and percentage activity index (PAI) calculated for the pathogens.

Microorganism	CIP	ZOI	MIC	MBC	AMI	PAI
	ZOI (mm)	(mm) ± SD	(μg/mL) ± SD	(μg/mL) ± SD		
*Bacillus cereus*	13.8	14 ± 1.10	1.75 ± 0.08	1.75 ± 0.08	1.01	101
*Bacillus subtilis 168*	14.5	15 ± 0.85	1.50 ± 0.07	1.50 ± 0.07	1.03	103
*Micrococcus luteus*	17.3	17 ± 1.25	1.25 ± 0.04	1.25 ± 0.04	0.98	98
*Pseudomonas aeruginosa*	12.5	12 ± 0.55	2.50 ± 0.20	2.50 ± 0.20	0.96	96
*Salmonella typhimurium*	13.0	12 ± 0.55	2.75 ± 0.07	2.75 ± 0.07	0.92	92

**Table 4:** High performance liquid chromatography (HPLC) mobile phase gradient.

Time/min	Con B/%
0	3
4	8
53	35
57	100
60	10

**Table 5:** The detected protein components by liquid chromatography-mass spectrometry (LC-MS).

Accession	Exp. q-Value:	Coverage	PSMs	Protein Groups	MW (kDa)
	Combined	(%)			
**A0A1Q9FQ60**	0	68	34	1	24.7
**A0A1Q9FUJ0**	0	60	117	1	22.5
**A0A1Q9FEN7**	0	42	6	1	22.2
**A0A1Q9FY79**	0	26	9	1	56.3
**Q65HB3**	0	18	7	1	22.5
**A0A1Q9FQG6**	0	13	5	1	34.7
**A0A1Q9FZC9**	0	4	5	1	154.2
**A0A1Q9FDF5**	0	22	5	1	30.6
**A0A1Q9FXL7**	0	26	5	1	16.6
**A0A1Q9G001**	0	38	45	1	15.6
**A0A1Q9FGV8**	0	12	3	1	27.7
**A0A1Q9FFF3**	0	20	3	1	20.7
**A0A1Q9FEE0**	0	8	3	1	31.2
**A0A1Q9G2J3**	0	16	7	1	20.6
**A0A1Q9G213**	0	6	2	1	31.9
**A0A1Q9FYJ5**	0	11	3	1	39.6
**A0A1Q9FEA7**	0	31	2	1	14.5
**A0A1Q9FYR5**	0	10	4	1	43.8
**A0A2U9VQ97**	0	11	4	1	20.7
**A0A1Q9FMJ6**	0	11	2	1	15.8
**A0A1Q9FXQ7**	0	13	2	1	37.4
**A0A1Q9FR47**	0	10	1	1	20.1
**A0A1Q9FZF7**	0	28	2	1	10.3
**A0A1Q9FZX5**	0	33	2	1	8.5
**A0A1Q9FXQ3**	0	7	2	1	33.3
**A0A1Q9FIV4**	0	1	2	1	154.7
**A0A1Q9FSE5**	0	9	1	1	17.1
**A0A1Q9G228**	0	5	1	1	35.3
**A0A1Q9FME6**	0	11	1	1	14.7
**Q65FN0**	0	11	1	1	15.3
**A0A1Q9FQE4**	0	12	1	1	11.4
**A0A1Q9FIX4**	0	3	1	1	36.9
**A0A1Q9G1Z5**	0	3	1	1	45.1
**A0A1Q9FRD3**	0	4	2	1	48.6
**A0A1Q9FIY1**	0	12	2	1	25.6
**A0A1Q9FJ07**	0	5	2	1	65.4
**T5HNB6**	0	2	1	1	51.7
**A0A1Q9FIQ8**	0	3	1	1	35
**A0A1Q9FDA4**	0	2	1	1	53
**A0A1Q9FMH1**	0	4	1	1	24.1
**A0A1Q9FLM0**	0	4	1	1	25.4
**A0A1Q9FSI6**	0	6	1	1	14.9
**A0A1Q9FLW4**	0	8	1	1	17.3
**A0A1Q9FZZ7**	0.015	2	1	1	50.3
**A0A1Q9G206**	0.015	6	1	1	17.7
**A0A1Q9FUC1**	0.015	6	1	1	15.2
**A0A1Q9FLZ2**	0.015	6	1	1	23.2
**A0A1Q9FEC9**	0.014	7	1	1	13.9
**A0A1Q9FXG7**	0.014	4	1	1	25.3
**A0A1Q9FY38**	0.014	2	1	1	35.8
**A0A1Q9FGR1**	0.014	12	1	1	15.9
**A0A1Q9FGD6**	0.013	9	1	1	26.2
**A0A1Q9G1N8**	0.013	7	1	1	17.5
**G4XU89**	0.013	3	1	1	31.8
**A0A1Q9FYH0**	0.013	2	1	1	38.1
**A0A1Y0YIP0**	0.012	3	1	1	48.1

## Data Availability

Data will be made available on request.

## Supplementary Materials

Supplementary material related to this article can be found online at: https://www.mdpi.com/article/10.3390/molecules28031058/s1.

## Sample Availability

The samples that support the findings of this study have been deposited, to the lab of Beijing Advanced Innovation Center for Food Nutrition and Human Health, Beijing Technology & Business University (BTBU), Beijing 100048, China.
