# Correction: Naveed et al. Purification, Characterization and Bactericidal Action of Lysozyme, Isolated from *Bacillus subtillis* BSN314: A Disintegrating Effect of Lysozyme on Gram-Positive and Gram-Negative Bacteria. *Molecules* 2023, *28*, 1058

**DOI:** 10.3390/molecules30244809

**Published:** 2025-12-17

**Authors:** Muhammad Naveed, Yadong Wang, Xian Yin, Malik Wajid Hussain Chan, Sadar Aslam, Fenghuan Wang, Baocai Xu, Asad Ullah

**Affiliations:** 1 Beijing Advanced Innovation Center for Food Nutrition and Human Health, Beijing Technology & Business University (BTBU), Beijing 100048, China; naveedbtbu@gmail.com (M.N.); wangyadong@btbu.edu.cn (Y.W.); yinxian@btbu.edu.cn (X.Y.); asadbahi2016@gmail.com (A.U.); 2 School of Light Industry, Beijing Technology & Business University (BTBU), Beijing 100048, China; 3 Department of Chemistry, Faculty of Science, Federal Urdu University of Arts, Science and Technology, Campus Gulshan-e-Iqbal, Karachi 75300, Pakistan; chanwajid@gmail.com; 4 Department of Biological Science, University of Baltistan, Skardu 16400, Pakistan; sadaraslam@gmail.com; 5 Food and Marine Resources Research Center, Pakistan Council of Scientific and Industrial Research Laboratories Complex, Karachi 75280, Pakistan

## Error in Figure

In the original publication, an error was identified in Figure 7 as published [1]. During the peer-review process, one reviewer noted that Figure 7 was not sufficiently clear and recommended cropping the image. After cropping, the figure could have been perceived as manipulated; therefore, we submitted the original SDS figure instead.

The corrected Figure 7 and the updated figure caption appear below. The authors confirm that the scientific conclusions remain unchanged. This correction was approved by the Academic Editor. The original publication has also been updated.

## Figures and Tables

**Figure 7:**
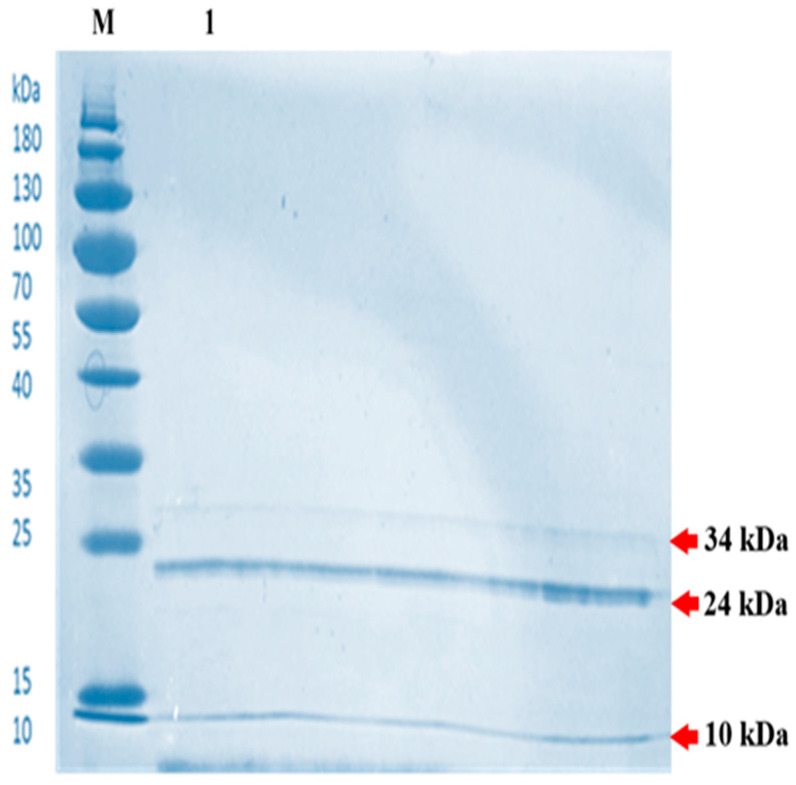
Analysis of *Bacillus subtilis* BSN314 lysozyme by sodium dodecyl sulfate-polyacrylamide gel (SDS-PAGE); Separation via SDS-PAGE resolves the purified protein into three distinct bands corresponding to molecular masses of 34 kDa, 24 kDa, and 10 kDa. This banding profile confirms the successful extraction and purification of the lysozyme to its fundamental monomeric form. Lane M contains the standard protein molecular weight marker; lane 1 contains the purified BSN314 lysozyme sample.
