# Supplementary material for: Purification, Characterization and Bactericidal Action of Lysozyme, Isolated from Bacillus subtillis BSN314: A Disintegrating Effect of Lysozyme on Gram-Positive and Gram-Negative Bacteria
Source: Molecules. 2023 Jan 20;28(3):1058. doi: 10.3390/molecules28031058 (PMC9919333; doi:10.3390/molecules28031058)

Supplementary Data for molecules-2148470

MIC/MBC Supporting Materials

Bacterial Cultures

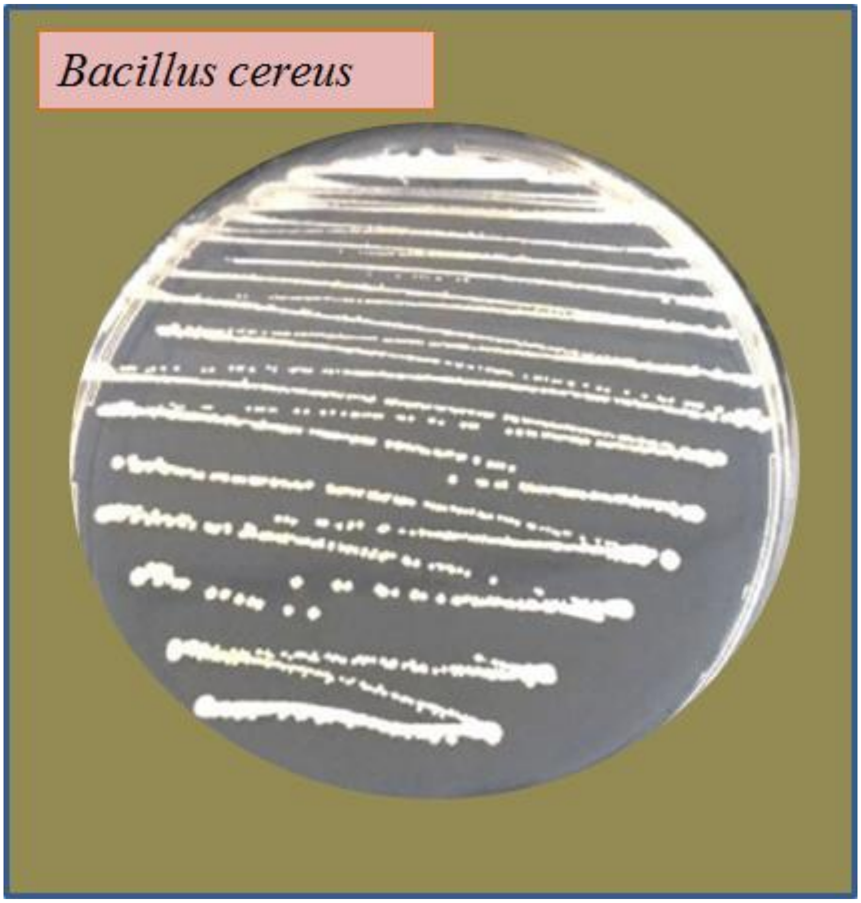

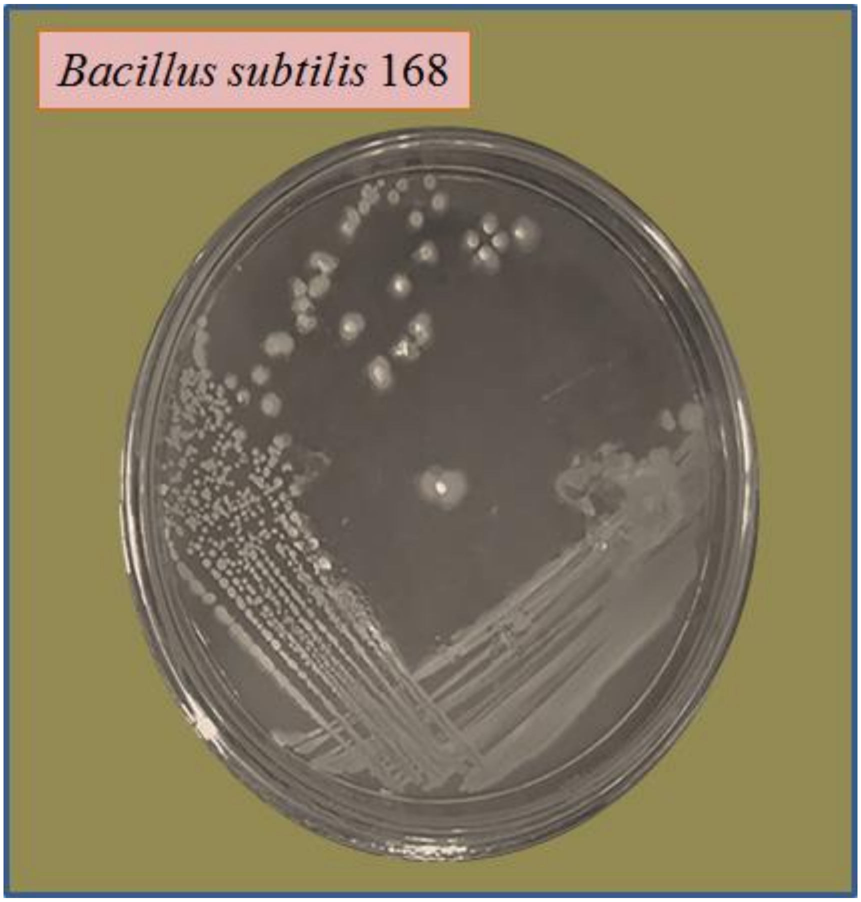
Bacillus subtilis 168

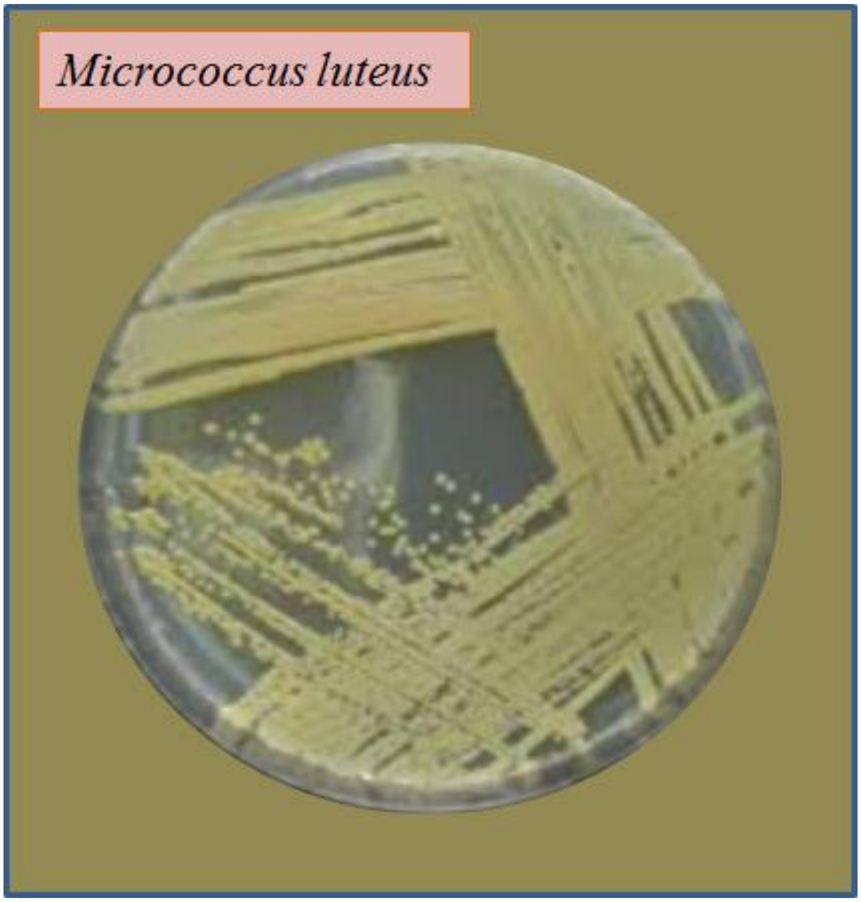
Micrococcus luteus

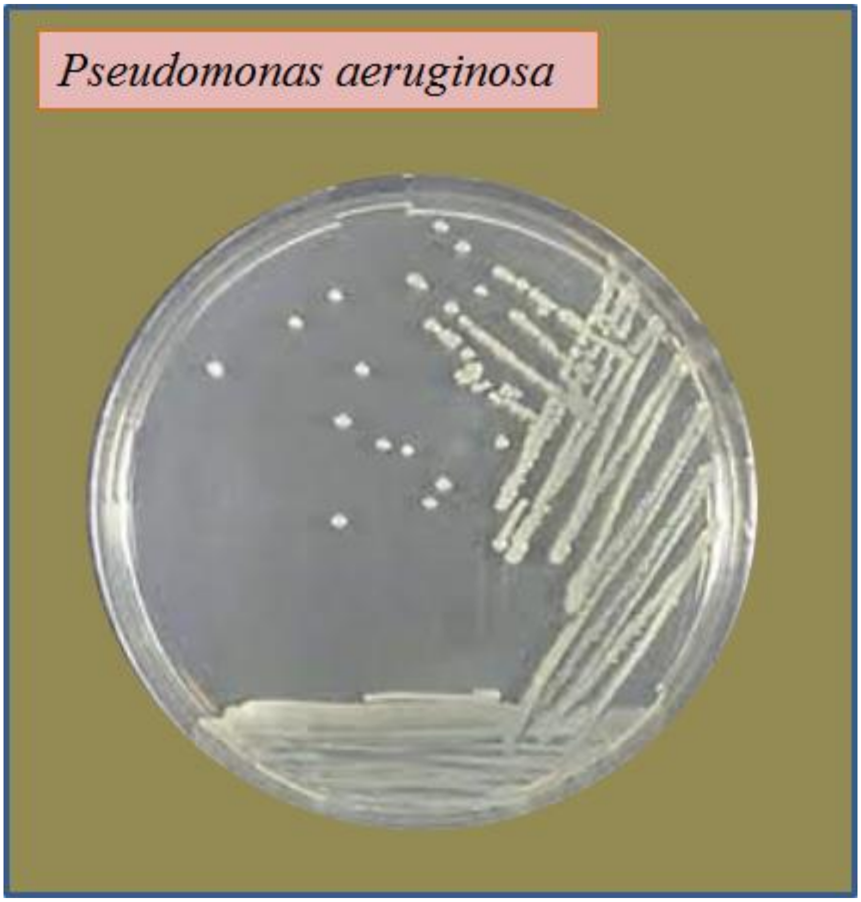

Pseudomonas aeruginosa

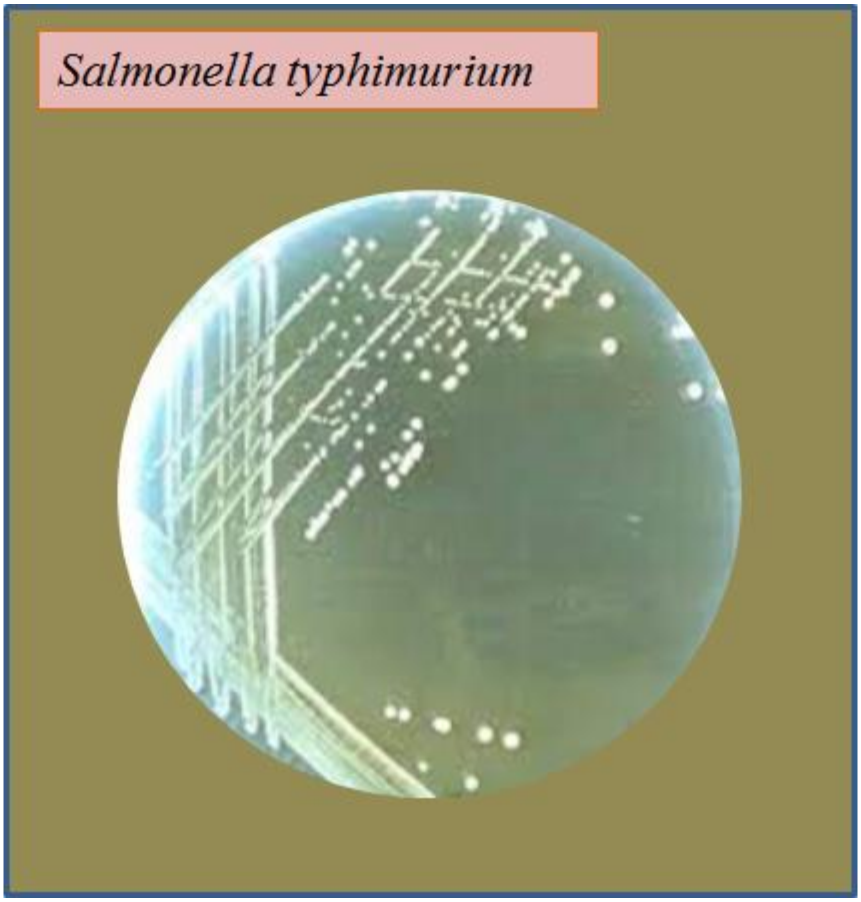
Salmonella typhimurium

Culture in Broth Media with Lysozyme Showing No Growth

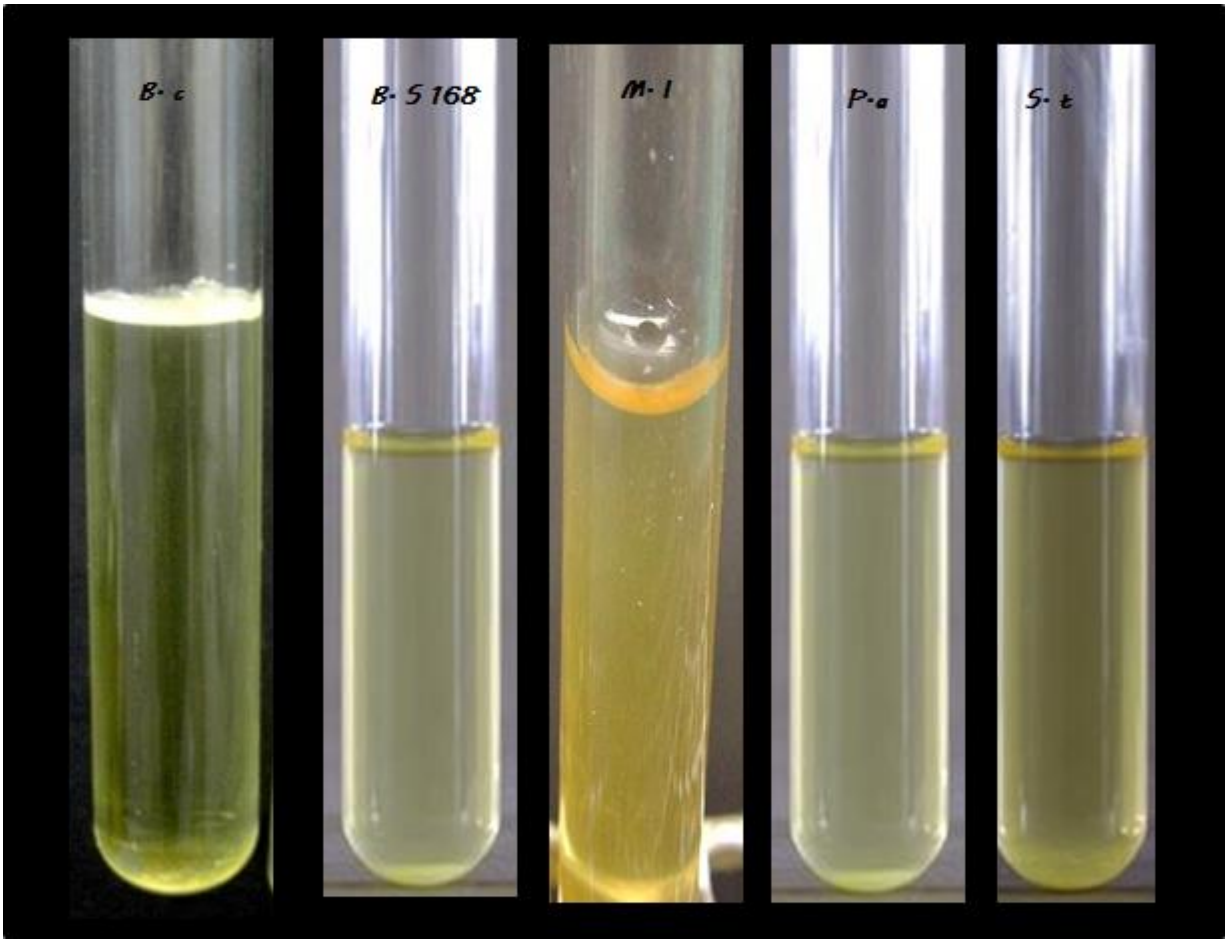

Shifting the Material From the Broth Media To the Agar Media Showing No Growth

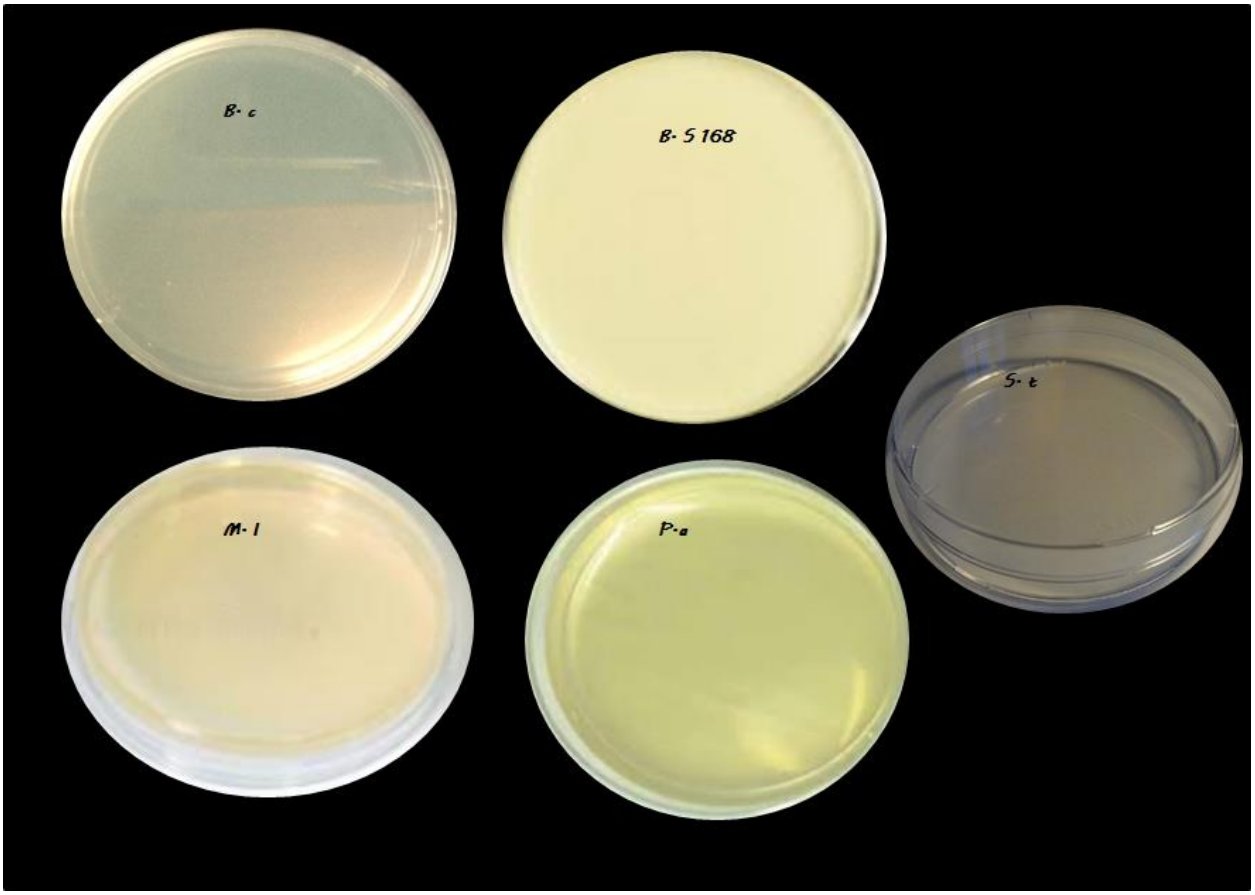

Supplement: Supplementary file 1 [file molecules-28-01058-s001.zip › molecules-2148470-supplementary.pdf]
